# Spatial clusters of dominant lineages of uropathogenic Escherichia coli in a community dwelling patient population

**DOI:** 10.21203/rs.3.rs-6350015/v1

**Published:** 2025-05-07

**Authors:** Cheyenne Belmont, Pushkar Inamdar, Salma Shariff-Marco, Amina Gul, Alison J. Huang, Henry F. Chambers, Eva Raphael

**Affiliations:** University of California, San Francisco; University of California, San Francisco; University of California, San Francisco; Khyber Medical College; University of California, San Francisco; University of California, San Francisco; University of California, San Francisco

## Abstract

**Introduction:**

Antimicrobial resistance (AMR) is a major public health concern, especially in the clinical management of urinary tract infections (UTIs). While use of antimicrobial agents selects for AMR bacterial strains, it remains unclear if this factor alone drives the prevalence of UTIs caused by AMR uropathogenic *Escherichia coli* (UPEC) in community settings. Local prevalence of AMR UTIs may be largely influenced by spatial clusters of already-resistant sequence types within a community rather than by the initial selection of resistant strains by antimicrobial agents. The goal of this study is to examine geospatial clustering of UTI by common AMR UPEC ST lineages.

**Methods:**

We collected 551 UPEC isolates from patients receiving care in a San Francisco public healthcare system from April to September 2019. Isolates underwent multiplex PCR for rapid identification of pandemic UPEC STs (ST69, ST73, ST95, ST131) and were linked with electronic health records data. We conducted Global Moran’s I and Local Moran’s I to detect spatial clusters of each pandemic ST lineage.

**Results:**

Forty five percent of UPEC isolates (N = 247) were identified as pandemic ST lineages. ST131 comprised 72 (29%) of the pandemic ST lineages and contributed the most multidrug resistant isolates (resistant to ≥ 3 classes of antibiotics) (N = 29). Spatial clusters of ST95, ST131 and ST69 (p < 0.001, p < 0.001, p = 0.008, respectively) were identified.

**Conclusion:**

We found spatial clusters of community-onset bacteriuria caused by predominant ST lineages, suggesting common-source outbreaks. This novel approach may inform future surveillance efforts to reduce community transmission of AMR UPEC and provides the basis for future investigations of environmental risk factors for AMR UTI.

## INTRODUCTION

Community-onset urinary tract infections (UTIs) are exceedingly common infections worldwide. An estimated 150 million people develop UTIs globally every year.^1^ These infections are associated with significant clinical and economic burdens to patients and healthcare systems.^1^ Antimicrobial resistance (AMR) is a critical challenge in the clinical management of UTIs. In 2019, UTIs were found to be the 4th leading cause of death associated with bacterial AMR.^2^ While an increase in multidrug-resistant (MDR) UTIs has long been recognized in hospital settings, evidence of an increase in the prevalence of MDR UTIs in community settings is concerning.^2,3^ It is unclear whether such an increase is due to antibiotic selective pressures alone or increase in prevalence and transmission of already resistant uropathogenic bacteria.

While *Escherichia coli* (*E. coli*) remains the primary cause of community-onset UTIs, this taxonomic group represents a complex and diverse range of organisms with significant variations between strains. From healthy human commensal flora to those associated with UTIs and gastrointestinal illnesses, the genetic diversity within this species is wide-ranging. Indeed, only 39.2% of predicted proteins are shared across enterohemorrhagic, uropathogenic, and commensal *E. coli* strains.^4^ Thus, meaningful epidemiological grouping is needed to understand how different sequence types (ST) may impact health. Molecular techniques, such as multilocus sequence typing (MLST), enable the rapid identification of new modes of transmission for infectious agents and facilitating the detection of strain-specific outbreaks within endemic disease patterns. This is especially true for UTIs which are often thought to represent sporadic events related to personal hygiene, sexual activity, or medical procedures like catheterization. However, through genotypic investigations using MLST, it has been found that about half of all community-onset UTI are caused by closely related *E. coli* lineages.^5–7^ This suggests possible common-source exposures to already resistant UPEC. Several studies have identified spatial clusters of AMR Enterobacteriaceae infections in the community, which may be representative of such common-source exposures.^8–11^ It is currently unknown if specific *E. coli* ST cluster geographically as well. If they do cluster, this will further support the hypothesis that seemingly sporadic AMR UTI events are the result of transmission dynamics and possibly associated to environmental factors, such as water quality, sanitation, and food exposures.

In this cross-sectional study, we collected clinical urine isolates routinely collected as part of medical care from April to September 2019. We identified *E*. *coli* lineages and investigated spatial patterning of prevalent *E. coli* lineages causing community-onset bacteriuria. By understanding how *E. coli* lineages causing community-onset bacteriuria are spatially distributed within a community, we can enhance our understanding of AMR UPEC transmission patterns and possibly identify possible local outbreaks and environmental exposures.

## MATERIALS AND METHODS

### Isolate collection

This is a cross-sectional study assessing the geographic distribution of uropathogenic *E. coli* STs. Our study is based in a large safety-net public hospital in San Francisco, the San Francisco General Hospital and the San Francisco Health Network, that serves an estimated 100,000 patients annually. The hospital microbiology laboratory conducts clinical testing for 15 associated clinics and a local chronic care facility, located in 14 San Francisco neighborhoods. We collected all Gram-negative bacterial isolates from clinical urine cultures sent for routine testing from April 2019 to September 2019 (N = 1007) processed at the hospital microbiology laboratory.

Electronic medical record (EMR) data, abstracted by the UCSF CTSI data abstraction services, was linked to clinical isolate data. Here, we include urine cultures from patients with suspected UTI and asymptomatic bacteriuria. We define community-onset bacteriuria episodes caused by *E. coli* as cases in which a urine culture was obtained from an outpatient clinic or emergency department, or within 48 hours of inpatient admission, and yielded an organism identified as *E. coli*.

The patient demographic characteristics and comorbidity data were extracted from the EMR included patient geocoded address as of 2019, age at time of culture, sex (male or female), self-reported race and ethnicity (Asian American or Pacific Islander, Black, Latine, White, or other/ declined to state), and preferred language spoken (Mandarin and Cantonese, English, Spanish, other or not stated). Comorbidities were evaluated based on the previous 5 years of EMR ICD-9 and ICD-10 codes and an unweighted Charleston Comorbidity Index (CCI) score was calculated.^12^ This study was approved by the UCSF Committee on Human Research (IRB number 19–27233) and the SFGH Research Committee.

### Speciation and antibiotic susceptibility testing

Bacterial isolates were collected from the hospital microbiology laboratory on blood agar purity plates and we further sub-cultured isolated on MacConkey and Blood Agar Biplates. The biochemical profile of urine bacterial isolates was confirmed by the hospital microbiology laboratory based on current Clinical and Laboratory Standards Institute (CLSI) guidelines.^13^ Isolates were speciated with API 20E (bioMérieux, Durham, NC) for fermenters or API 20NE for non-enteric bacteria. Indole testing was conducted as secondary confirmation of presumptive *E. coli* in our laboratory. The hospital microbiology laboratory performs antimicrobial susceptibility testing (AST) using Microscan WalkAway Gram-negative panel and disk diffusion, with classification of resistance based on CLSI breakpoint standards.^13^ The microbiology laboratory classified extended-spectrum beta-lactamase producing *E. coli* (ESBL-*E. coli*) as an *E. coli* strain resistant to ceftazidime or cefotaxime and inhibited by clavulanic acid using broth microdilution, per 2016 CLSI guidelines.^13^ A multidrug resistant (MDR) isolate was defined by phenotypic resistance to at least 1 agent in ≥ 3 classes of antimicrobial agents used to treat UTI (β-lactams, fluoroquinolones, aminoglycosides, trimethoprim-sulfamethoxazole, and nitrofurantoin).^13^ Results reported as “intermediate resistance” were considered resistant in this study.

### DNA extraction and sequence typing

All bacterial DNA was extracted by freeze-boil method. *E. coli* sequence types (STs) 69, 73, 95, and 131 were identified by a validated multiplex polymerase chain reaction (PCR) yielding PCR products of expected sizes (Table S1).^14^ Gel electrophoresis was used to distinguish unique band sizes to identify *E. coli* sequence types.^15^

### Statistical and geospatial analysis

Key patient demographic and isolate characteristics were summarized with descriptive statistics, including frequencies and percentages for categorical data and mean values with maximum and minimum values for continuous data. All analyses were conducted in R 3.0.1. Charleston’s comorbidity index was calculated using the comorbidity package in R.^12^

All spatial analyses were conducted with ArcGIS Pro. Urine isolates from patients without San Francisco residential addresses or who did not meet the criteria of community-onset bacteriuria were excluded from analyses. We conducted separate spatial analyses to identify geographic clusters of the 4 major pandemic *E. coli* STs within San Francisco County. A kernel density heatmap was created to assess the community-onset bacteriuria patient distribution within San Francisco. The density of points at any given location is calculated by summing the contributions of all the kernel functions centered at data points in the vicinity of that location. Patient residential confidentiality was ensured by randomly substituting new point data within a fixed buffer diameter around the original address location. The potential for spatial heterogeneity or spatial patterns amongst each of the four lineages was assessed by Global Moran’s I based on Euclidean distance and inverse distance methodology, such that all patients have at least 1 neighbor. Global Moran’s I is a statistical measure used to determine the degree of spatial autocorrelation in a dataset. Spatial autocorrelation refers to the tendency of similar values to cluster together in geographic space. Global Moran’s I calculates a single value for an entire study area or dataset, which represents the overall degree of spatial clustering or dispersion in the dataset. The value of Global Moran’s I can range from − 1 (perfect dispersion) to + 1 (perfect clustering), with 0 indicating no spatial autocorrelation. A positive value of Global Moran’s I indicates that values of the variable being analyzed are clustered together in space, while a negative value indicates that they are dispersed.^16^

Cluster identification was conducted through Aselin Local Moran’s I, based on Euclidean distance method and fixed distances. Bond threshold was determined by iteratively testing distances beginning at the average distance between cases to maximize spatial autocorrelation. Local Moran’s I, also known as the local indicator of spatial association (LISA), is a statistical measure used to identify spatial clusters of high or low values for a specific variable within a study area or dataset. Local Moran’s I is a localized version of Global Moran’s I, which calculates the degree of spatial autocorrelation across the entire dataset. Local Moran’s I calculates a separate value for each individual unit or location within the study area, which represents the degree to which that unit is surrounded by other units with similar or dissimilar values. Like Global Moran’s I, Local Moran’s I can range from − 1 to + 1, with positive values indicating clustering of similar values and negative values indicating dispersion of similar values. Local Moran’s I is useful in identifying areas of high or low spatial clustering of a specific variable.^16^

Choropleth maps were generated by conducting a spatial join of cluster locations within San Francisco neighborhood boundaries defined in 2006 by the Mayor’s Office of Neighborhood Services and colored to visually display the number of high-high (HH) clusters and spatial low-low (LL) cluster of each dominant lineage within San Francisco.^17,18^ In examining the spatial distribution of a particular genetic UPEC ST lineage, a HH cluster would indicate a group of locations where the lineage is highly prevalent compared to other lineages including those that are not pandemic lineages, while a LL cluster would indicate a group of locations where the lineage is rare or absent compared to other lineages. Sensitivity analyses were conducted by adjusting for a false discovery rate within Local Moran’s I.

## RESULTS

### Patient demographic characteristics

Among the study population (N = 551), only 40 isolates (7%) came from male patients and the median patient age was 48 (Table 1). Most patients identified as Latine (36.3%) and the most common preferred languages were English (37.2%), followed by Spanish (25.4%). The average CCI value of all patients was 3.44, patients whose urine grew ST73 had the lowest CCI (2.50) and those whose urine grew ST69 had the highest CCI (3.7). Only 43 patients (7.8%) were diagnosed with a prior UTI within the 5 years of the current episode.

### Prevalence of antimicrobial resistance by sequence type

Of the 551 UPEC isolates in the study, 247 (45%) were identified as pandemic lineages (Table 2). ST131 was the most common lineage representing 72 (29%) of the pandemic STs and contributing the majority of MDR isolates (85%) and ESBL isolates (81%). The most pan-susceptible lineage was ST95; 39 (56%) isolates from that lineage were susceptible to all tested antibiotics. Resistance to fluoroquinolones was rare in all lineages, except for ST131, where 47% of isolates demonstrated resistance to fluoroquinolones. The only lineage among pandemic lineages that demonstrated resistance to nitrofurantoin was ST131 (3%).

### Spatial analyses

**Figure 1:**
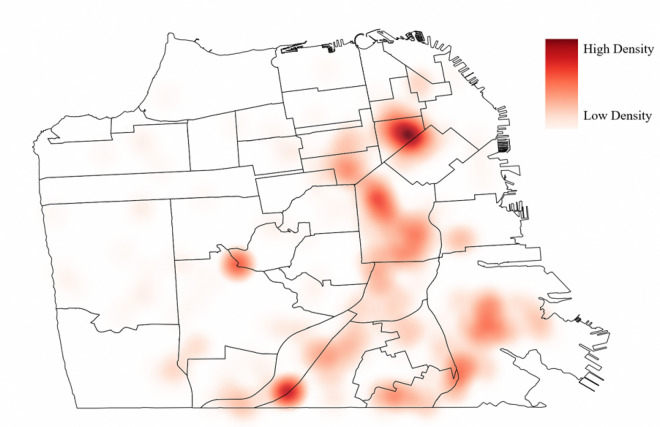
Distribution of CA-UTI within San Francisco caused by *E. coli*. Note: Heat map was created using magnitude-per-unit area from point features using a kernel function within the kernel density tool in ArcGIS Pro.

Of the 551 *E. coli* isolates, 10 patient addresses could not be geolocated and 19 did not meet community-onset bacteriuria inclusion criteria. Additionally, 32 patient addresses were located outside of San Francisco County and were excluded from the analysis. The distribution of patient addresses within San Francisco was visualized in a kernel density heat map (Fig. 1). Map areas of high density of patients with community-onset bacteriuria are represented by darker colors and areas of low density are represented by lighter colors. The outcome of the Global Moran’s I tests of ST95, ST131 and ST69 showed evidence of spatial heterogeneity, or spatial clusters (p = 0.001, p = 0.001, p < 0.001, respectively) within San Francisco County (Table 3). There was an uneven distribution of various concentrations of each ST within San Francisco, warranting further cluster resolution. Results of Local Moran’s I further discerned HH and LL clusters of ST95 and HH clusters of ST131 and ST69 (Table 3). When adjusting for false discovery rate, we detected two clusters of ST69 and no clusters of other STs.

**Figure 2:**
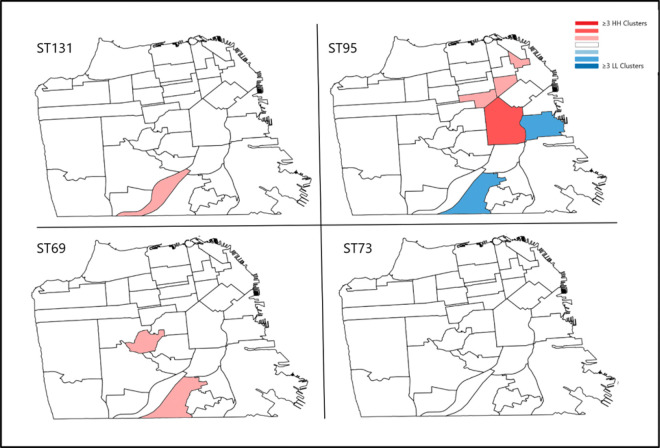
Distribution of spatial clusters of dominant lineages of UPEC within San Francisco Clusters were detected using local Moran’s I applying Euclidian distances without adjustment of FDR. Clusters identified were aggregated to neighborhood features, the sum of which are display in a choropleth map. Red shades denote number of clusters identified and blue shades indicate number of outliers detected in each neighborhood.

A choropleth map (Fig. 2) exhibits the presence of HH clusters and LL clusters with red and blue color ramps displaying clusters of each pandemic lineage as detected by Local Moran’s I.

## DISCUSSION

Community transmission of AMR UTI is a critical public health concern that warrants improved and local surveillance. Geographic information systems (GIS) have been commonly used to analyze and describe the geospatial distribution of many diseases in recent decades, especially infectious disease. Understanding spatial disease distribution and the potential of spatial clustering can provide insight into disease transmission, potential exposure sources, and disease reservoirs. Here, we leverage molecular biology data with EMR data to characterize the spatial distribution of uropathogenic *E. coli* STs, which may suggest patterns of disease transmission. Here, we found that 70% of bacteriuria episodes in a large safety-net healthcare system in San Francisco were caused by *E. coli*, with half belonging to 4 distinct lineages (ST95, ST69, ST131, and ST73). We identified spatial clusters of ST69, ST 95, and ST131, which indicates the possibility of common-source exposures to these lineages. Additionally, lineage ST131 was strongly associated with AMR, while ST95 was pan-susceptible, as reported in other studies.

To date, there is some evidence of spatial clustering of community-onset AMR UTI, but no study has established clustering of UPEC lineages. In Brazil and in the West of Ireland, neighborhood-level clusters of fluoroquinolone-resistant *E. coli* causing community-onset UTI were identified. Geospatial mapping of resistant *E. coli* isolates revealed that most AMR isolates clustered in urban regions.^19, 20^ These studies focused on how prescribing practices in these areas may be associated with these clusters of resistant phenotypes. However, our work is the first to demonstrate spatial clusters of already resistant lineages. This may play a major role in the distribution of community-onset AMR UTI independent of antibiotic prescribing patterns.

This study employed a cross-sectional study design which provides an opportunity to assess the prevalence of AMR *E. coli* causing bacteriuria and circulating sequence types. To our knowledge, this is the first report of spatial clusters of specific uropathogenic STs, demonstrating distinct variation in spatial patterns of ST prevalence. Possible transmission pathways include person-to-person exposures of UPEC, or dissemination of UPEC lineages from specific point source exposures. It may be that these bacteria are acquired from contaminated food products or other external sources within the built environment (e.g., water, environment) [18–24].^18,21–27^ A recent systematic review found that ESBL-producing *E. coli* belonging to the same lineages (ST131, ST69, ST73) were found in food sources, companion animals and water sources.^18^ Recently, a phylogenetic analysis and plasmid interrogation of ST131, recovered from poultry products, was found to be closely related to ST131 isolated from humans residing in the same region.^27^

Lineage ST131, which comprises 29% of our collection, has long been a lineage of concern, as it is strongly associated with ESBL phenotype and MDR. This is consistent prior reported that ST131 contributes 85% of MDR *E. coli*.^10^ Lineage ST95, conversely, has a documented propensity for remaining drug susceptible.^6–8^ In our collection, 56% of ST95 isolates were found to be pan-susceptible. Thus, the geographic distribution and dissemination of these lineages may have major implications for the transmission of AMR community-onset bacteriuria.

A major strength of this study is its ability to leverage linkages between bacterial genotype and patient EMR data to find evidence of lineage-specific geographic disease clusters. Our analysis relies on patient residential address to geolocate cases; however, a limitation of this study is its ability to capture disease distribution and transmission as it occurs in workplaces, schools, community venues, residences of close contact, and other settings. We examined the sensitivity of our Local Moran’s I results by additionally adjusting for a false discovery rate, which resulted in the loss of some, but not all clusters. The application of GIS methods within molecular epidemiological datasets is often limited by the restriction of feasible sample sizes. We believe that the decrease in clusters identified from 7 to 2 is likely due to small sample size, but, overall, the results of the Local Moran’s I analyses demonstrate that our findings are robust. Another limitation is that spatial analyses were restricted to patients with residential addresses and did not include those experiencing homelessness. Lastly, our analyses are limited to urine cultures sent routinely for testing, there may be some selection bias present due to the clinical presentation of the patient and the individual practice of the clinician.

## CONCLUSION

This investigation harnesses molecular and spatial epidemiology methods to identify spatial clusters of uropathogenic bacterial lineages ST69, ST95, and ST131. Here, bacteriuria cases exhibited spatial clustering throughout San Francisco. This highlights the potential of AMR lineages, like ST131, to occur in outbreaks outside of hospital settings. Future research should prioritize investigation of spatial heterogeneity within UPEC lineages causing community-onset bacteriuria alongside other potential community level risk factors - particularly those related to built-environments and exposures other than antibiotics which may contribute to the increasing prevalence of AMR UTI.

## Figures and Tables

**Table 1:** Demographic and health characteristics of patients with UPEC infection by dominant sequence type.

	All isolates (N = 551)	ST95 (N = 70)	ST131 (N = 72)	ST69 (N = 53)	ST73 (N = 52)
Age median (max, min)	48 (1, 95)	52 (20, 95)	53 (17, 89)	36 (3, 88)	40 (6, 75)
Sex (male)	40 (7.3%)	7 (10%)	6 (8.3%)	3 (5.7%)	6 (11.5%)
Race/ Ethnicity					
White	57 (10.3%)	3 (4.3%)	15 (20.8%)	3 (5.7%)	5 (9.6%)
Black	36 (6.5%)	6 (8.6%)	8 (11.1%)	2 (3.8%)	3 (5.8%)
Asian/Pacific Islander	65 (11.8%)	20 (28.6%)	5 (6.9%)	4 (7.5%)	8 (15.4%)
Latine	200 (36.3%)	25 (35.7%)	19 (26.4%)	21 (39.6%)	19 (36.5%)
Other/ Declined to state	358 (64.9%)	54 (77.1%)	47 (65.2%)	30 (56.6%)	35 (67.3%)
Preferred Language					
English	205 (37.2%)	32 (45.7%)	36 (50.0%)	13 (24.5%)	19 (36.5%)
Spanish	140 (25.4%)	18 (25.7%)	13 (18.1%)	17 (32.1%)	12 (23.1%)
Mandarin & Cantonese	22 (4.0%)	5 (7.1%)	2 (2.8%)	1 (1.9%)	3 (5.8%)
Other	19 (3.4%)	2 (2.9%)	1 (1.4%)	3 (5.7%)	2 (3.8%)
Not Stated	165 (29.9%)	13 (18.6%)	20 (27.8%)	19 (35.8%)	16 (30.8%)
Previous UTI	43 (7.8%)	8 (11.4%)	13 (18.1%)	1 (1.9%)	4 (7.7%)
Recurrent UTI	18 (3.3%)	1 (1.4%)	8 (11.1%)	0 (0%)	2 (3.8%)
*Co-morbidities*					
Diabetes	13 (2.4%)	4 (5.7%)	2 (2.8%)	0 (0%)	1 (1.9%)
Prior Antibiotics (6 mo.)	33 (6.0%)	4 (5.7%)	10 (13.9%)	1 (1.9%)	4 (7.7%)
Malignancy	17 (3.1%)	0 (0%)	8 (11.1%)	0 (0%)	3 (5.8%)
CCI* mean (SD)	3.44 (1.13)	2.63 (2.26)	3.23 (1.01)	3.70 (1.03)	2.50 (2.52)

Patient characteristics were extracted from eMRs: age at time of culture; sex (male or female); race and ethnicity (Asian or Pacific Islander, Black, Latine, White, or other/ declined to state); andpreferred language spoken (Mandarin and Cantonese, English, Spanish, other or not stated).Comorbidities were evaluated using 5 years of ICD-9 and ICD-10 codes.

* Unweighted CharlestonComorbidity Index score, mild with CCI scores 1–2; moderate with CCI scores of 3–4; and severe,with CCI scores ≥ 5

**Table 2:** Antimicrobial susceptibility by dominant sequence type.

Sequence Type	Number of episodes caused by susceptible isolates	Number of episodes caused by antimicrobial resistant isolates (%)					
		Ampicillin	Nitrofurantoin	Trimethoprim-sulfamethoxazole	Fluoroquinolones	ESBL	MDR
ST95	39 (56%)	23 (33%)	0 (0%)	38 (53%)	1 (1%)	0 (0%)	1 (1%)
ST131	13 (18%)	55 (75%)	2 (3%)	38 (53%)	34 (47%)	22 (31%)	29 (40%)
ST69	13 (18%)	36 (68%)	0 (0%)	30 (57%)	3 (6%)	4 (8%)	0 (0%)
ST73	17 (33%)	32 (62%)	0 (0%)	13 (25%)	3 (6%)	1 (2%)	4 (8%)

Antimicrobial susceptibility testing was performed with Microscan and disk diffusion methods, and ESBL status was confirmed with reports of resistance based on CLSI breakpoint guidelines. MDR is defined as resistant to at least one agent in ≥3 classes of antibiotics.

Abbreviations: ESBL: extended-spectrum beta-lactamase, MDR: multidrug resistant

**Table 3:** Global and Local Moran’s I analysis to detect spatial heterogeneity and local clusters of dominant lineages of UPEC.

	ST95	ST131	ST69	ST73
*Global Moran’s I*				
Moran’s index	0.0874	0.166	0.112	0.031
P-value	0.001	0.001	<0.001	0.2493
*Local Moran’s I*				
HH Clusters Detected	5	1	2	--
LL Clusters Detected	3	0	0	--
*Local Moran’s I FDR adjustment*				
HH Clusters Detected	0	0	2	--
LL Clusters Detected	0	0	0	--

Patterns of spatial heterogeneity were detected using Global Moran’s I, using Euclidian distances and inverse distance methodology. Spatial clusters were detected using Local Moran’s I with Euclidian distances and fixed distances. Additional sensitivity analysis was conducted with a false discovery rate adjustment.

## Data Availability

The datasets generated and/or analysed during the current study are available in the Zenodo repository (DOI: 10.5281/zenodo.15190784).
